# Supplementary material for: Presence of known feline ALMS1 and MYBPC3 variants in a diverse cohort of cats with hypertrophic cardiomyopathy in Japan
Source: PLoS One. 2023 Apr 18;18(4):e0283433. doi: 10.1371/journal.pone.0283433 (PMC10112785; doi:10.1371/journal.pone.0283433)
Supplement: Attachment — Submitted filename: Reply_to_comments.docx [file pone.0283433.s004.docx]

From Editor

>1. Please ensure that your manuscript meets PLOS ONE's style requirements, including those for file naming.

**Response:** Thank you for pointing this out and providing the link to PLOS ONE’s style guidelines. We have revised the manuscript according to the template.

>2. At this time, please address the following queries

**Response:** We have responded to all four queries. Please see the cover letter.

>3. We noted in your submission details that a portion of your manuscript may have been presented or published elsewhere.

>Please clarify whether this [conference proceeding or publication] was peer-reviewed and formally published.

**Response:** This paper was not previously peer-reviewed or published as either a conference proceeding or a journal publication. Therefore, there will be no duplication.

> 4. We note that you have included the phrase “data not shown” in your manuscript.

**Response:** We have added the necessary text and tables to the relevant parts of our manuscript and included the Sequence Read Archive ID of each data.

Reviewer 1

> The contents of the manuscript should be stated in an appropriate order. For example, limitations and conclusions are not listed where they should be. That makes the manuscript confusing. Also, the reviewer highly recommends the authors make subtitles for each subsection in the Discussion, and the first subsection should be the summary of findings in the present study. These modifications would make the manuscript easier to comprehend.

> The last paragraph of the Introduction contains the results of this investigation. The author should move these descriptions to the discussion section, or conclusions section (create it after the discussion section).

**Response:** Thank you for your suggestion. We followed your suggestion and have rearranged the text pertaining to limitations, by moving it to the appropriate sections ( Lines 292 to 296). We have included subsections in the Discussion and have rearranged the summary sentence (Lines 207 to 211).

> Whereas MYBPC3 p.A31P is an established genetic mutation in HCM, ALMS1 p.G3376R is not established (the association with HCM is just suggested). These two findings should not be listed in the same importance. The authors should state the clinical implication of these findings in each subsection, or in each paper.

**Response:** We agree with your suggestion. The clinical significance of our findings has been described in the Discussion section in Lines 227 to 230 and 232 to 236. We have also mellowed down the tone of the subsection by removing the sentence pertaining to the relationship between the *ALMS1* variant and HCM in American Shorthair.

> In the last sentence of page 16, the expression “genetic testing of MYBPC3 p.A31P in non-Maine Coon breeds is necessary for HCM diagnosis and to prevent the establishment of HCM-affected colonies”, seems too strong for the suggestion from results with a relatively small number of subjects. The author would be advised to alter the expression in an appropriate manner.

**Response:** We followed your advice and have revised the sentence accordingly (line 285-287).

> (Discussion, Page 14, Line 13) The author should add “Bell et al., 2012” into the Reference.

**Response:** Owing to a change in the text, Bell et al. 2012 was not cited.

> (Table 1) As per the manuscript, the MYBPC3 p.A31P in Munchkin is the variant that has never been found. Therefore, an asterisk should be attached next to the number “1”.

> (Table 3) There is no row about Minuet. As per the manuscript, one heterozygote ALMS1 variant was found in the 2 Minuet cats. If the author considers the ALMS1 variant in Minuet is worth reporting, it should be listed also in the Table.

**Response:** Thank you for your point of view. We have revised the content in the tables accordingly.

> Figure 1 seems not to have enough information. It should be transformed into another table, which enables the authors to fill out the absolute numbers of each zygosity in each group. They can also attach percentages next to each absolute number.

**Response:** Thank you for your suggestion. We attempted to present the content in Figure 1 as a new table. The same values are presented in Table 1 and Table 3; therefore, to avoid duplication, we have retained the figure. Hope you would agree with us.

Reviewer 2

> - Abstract

> You should check the variant name p.R830W.

**Response:** Thank you for pointing this out. We have revised this.

> - Introduction

> At the end of this section you describe your results and conclusion.

>You shouldn’t include your results in the introduction Section.

**Response:** The summary text was moved to the Discussion (Lines 207-211).

> - Methods

> The authors include 2 Minuet cats without a cardiac phenotype in “Phenotyping and selected animals” section, nevertheless the genetic results are not provided. The authors should include the genetics results of these cats in table 3.

**Response:** Thank you for pointing this out. We have revised Table 3 accordingly.

>In this section the authors must detail the statistical analysis used.

**Response:** We followed your suggestion and have included a subsection on statistical analysis to the Materials and Methods (Lines 132-136).

> - Results

>I suggest to the authors that they include a table with the main clinical variables of the cats studied such as breed, age, age of phenotype, gender, mm hypertrophy, events or arrhythmia.

**Response:** Thank you for your suggestion. We have described the main clinical variables in S2 Table.

>The genetic results could be integrated and summarized in a table that includes the frequency of the variants and the comparison between the different groups of animals.

**Response:** Thank you for your suggestion. We attempted to merge the tables. The resulting table was too information intensive due to the increased sample classifications and could be difficult to read and follow. Therefore, we have retained the tables in their original form. Hope you would agree with us.
