# Supplementary material for: Presence of known feline ALMS1 and MYBPC3 variants in a diverse cohort of cats with hypertrophic cardiomyopathy in Japan
Source: PLoS One. 2023 Apr 18;18(4):e0283433. doi: 10.1371/journal.pone.0283433 (PMC10112785; doi:10.1371/journal.pone.0283433)
Supplement: Attachment — Submitted filename: Reply_to_comments_ym.docx [file pone.0283433.s005.docx]

From Reviewer 1

> The aim of this study was removed from the whole manuscript. It should be retained in the last part of the introduction.

**Response:** Thank you for your suggestion. We retained the aim of our study in the last part of the introduction (Lines 80 to 82).

> The reviewer still recommends that the authors should state the conclusion at the end of the manuscript. It should the main message to the reader clearer.

**Response:** We agree with your suggestion. We added conclusion section in Lines 313 to 318.

> All the abbreviations in the supplemental tables should be spelled out in the footnotes.

**Response:** We followed your advice and added column name in S2 table (“y” and “m” in age column, and ACVIM in “Heart failure stage” column), and footnote to S3 table (SRA Run ID for Line 439-440).

＞Although the authors added the clinical information of subjects in the supplemental table 2, they did not describe it in either the results or the discussion in detail. They should describe the clinical characteristics or cardiac events specific to MYBPC3 p.A31p positive cases or ALMS1p.G3376R positive cases.

**Response:** Thank you for your suggestion. We added description regarding clinical information in results section (Lines 163 to 172).
